# Transfusion Transmissible Infections: Maximizing Donor Surveillance

**DOI:** 10.7759/cureus.3787

**Published:** 2018-12-28

**Authors:** Sara A Awan, Ayesha Junaid, Shafain Sheikh

**Affiliations:** 1 Hematology, Shifa International Hospital, Islamabad, PAK; 2 Immunology, Shifa International Hospital, Islamabad, PAK

**Keywords:** nat, hiv/aids, syphilis, malaria, hbv, hcv, blood transfusion service, blood donors

## Abstract

Introduction

The World Health Organization (WHO) recommends that all blood transfusion services must screen donated blood for human immunodeficiency virus (HIV) one and two, hepatitis B, hepatitis C and syphilis. A mandatory screening for malaria is also warranted in malaria endemic areas. Our study aimed at analyzing the prevalence and different diagnostic methods of screening transfusion transmitted infections (TTIs) in replacement and voluntary, non-remunerated donors in the blood bank of a tertiary care hospital in Islamabad, Pakistan.

Methods

The cross-sectional, descriptive study was conducted on 30,470 blood donors from July 2015 to October 2017, in the blood bank of a 500-bed teaching hospital in Islamabad. Initially all blood donors were screened for HIV one, HIV two, hepatitis B and hepatitis C by serological testing. The seronegative samples were further tested by nucleic acid amplification test (NAT). Malaria was screened using immuno-chromatographic antigen-detection tests, while treponema pallidum was screened by electrochemiluminescence immunoassay to detect treponema pallidum (TP) antibodies. All infected blood and blood products were discarded and donors were contacted. The donors were deferred from blood donation according to WHO guidelines. They were also counselled and referred to the infectious diseases clinic. The collected data was analyzed on IBM's statistical package for the social sciences (SPSS) version 21.

Results

The results revealed that amongst the 30,470 donors, 997 (3.27%) donors were found infected with one or more TTI while 29,473 (96.73%) donors were found safe. Individuals who tested positive on serology for hepatitis B were 322 (1.06%), hepatitis C were 392 (1.29%) and HIV were 49 (0.16%). The seronegative donors were tested by NAT. NAT on seronegative samples showed that 10 (0.03%) donors tested positive for hepatitis B virus deoxyribonucleic acid, while only three (0.01%) were positive for hepatitis C ribonucleic acid. No donor was found positive for HIV by NAT testing. Syphilis testing revealed a frequency of 228 (0.75%) positive results while only five (0.02%) donors were found infected with malaria.

Conclusion

The results testify that standardized blood component screening can save transmission of infections through blood transfusion. They also establish the superiority of NAT screening over serological tests in decreasing the residual risk of transfusion transmitted infections.

## Introduction

A blood transfusion service is required to provide a safe and timely supply of blood and blood products. While it is of key importance to enforce measures to prevent transmission of any infection through blood transfusion, it is also equally important to reduce the unnecessary deferral of healthy donors. According to the World Health Organization (WHO) Global Database on Blood Safety, 1.6 million units out of 92 million units blood donated worldwide is discarded due to the presence of markers for transfusion transmitted infections [1]. This elucidates the significance of implementing strategies to investigate donor's beforehand, thereby reducing the wastage of resources.

Nucleic acid amplification test (NAT) allows the identification of infections during window period as opposed to serological tests. This offers blood centers a much higher sensitivity for detecting blood-borne infections. Resource-limited countries in Africa, Asia, and Latin America have a high prevalence of blood-borne viral infections. Therefore these countries are likely to yield a significant number of window period donations. As a result NAT is expected to be more cost-effective in these countries as compared to developed countries [2].

## Materials and methods

The study was conducted at Shifa International Hospital which provides inpatient and outpatient services to a varied mix of patients. It is unique in the region for offering liver, renal, bone marrow and corneal transplant services. The blood bank of the hospital processes more than 15,000 donors annually. It offers serology and NAT for hepatitis B virus (HBV), hepatitis C virus (HCV), and human immunodeficiency virus (HIV). Malaria is screened by immuno-chromatographic antigen-detection tests. Syphilis is screened using an electrochemiluminescence immunoassay to detect treponema pallidum (TP) antibodies.

Our study spanned from July 2015 to October 2017. Ethical approval of the study was obtained from Shifa International Hospital's Institutional Review Board and Ethics Committee. The inclusion criteria consisted of all voluntary and replacement non-remunerated blood donors walking in during this study period. The lower age limit for donors was set at 18 years while the upper limit was set at 60 years. A minimum weight of 50 kg was required to donate 450 ml ± 10% blood. All the donors were educated about the donation process and a written, informed consent was acquired. Each donor filled a standardized donor questionnaire, documenting their demographics, past medical and surgical history, travel history, high-risk activities and possible exposure to infectious agents. This allowed for health and risk assessment. Donors were deferred strictly in accordance to WHO guidelines. Any individual with an active HBV infection or those with a history of infection in the past 12 months was deferred, while counselling them that they may be able to donate blood 12 months after recovery. All individuals with an active or past HCV infection were permanently deferred. Similarly, any person with a present or past clinical or laboratory evidence of HIV was permanently deferred. Individuals diagnosed with syphilis were permanently deferred from blood donation as well. Malaria-infected patients were deferred for six months after completion of treatment and full recovery, whichever was longer.

Prospective donors were provided with materials for donor education and adverse donor reactions. Physical examination including height, weight, and vital statistics was documented for all donors. The minimum cut off for hemoglobin was set at 12.5 g/dl for females and 13.5 g/dl for males.

Each donor provided one 5-ml blood sample in serum separator tube for serological tests. Another 3-ml blood sample in EDTA (ethylenediaminetetraacetic acid) tube was collected for NAT and malaria screening. Serum samples were screened on a fully automated immunoassay analyzer for HBsAg, anti-HCV antibodies and HIV 1/2 antigen-antibody combination assay. Only the seronegative donor samples were subjected to NAT in a minipool of 6 units. Food and Drug Administration (FDA)-approved triplex NAT system using a multiplex polymerase chain reaction kit was used. Automated specimen pooling was performed on an automated pooling station. Once a pool was found reactive, a discriminatory NAT on the same system was performed in individual-donation NAT (ID-NAT) format to identify the reactive donor sample within the pool as well as to identify the individual virus.

All the reactive donors were contacted and notified the results maintaining patient confidentiality. They were referred to the infectious diseases clinic for further management. The collected data was analyzed on IBM's statistical package for the social sciences (SPSS) version 21.

## Results

The results revealed that in a period of 27 months (August 2015 to October 2017) 30,470 asymptomatic donors were screened for TTIs. Amongst these, 997 (3.27%) donors were found infected with TTIs and deferred from donating blood while 29,473 (96.73%) were found safe. On the practical front, NAT saved 52 potentially infected components being released for transfusion in our blood transfusion service, over a period of 27 months.

In the first round all donors were screened for HBV, HCV and HIV by serological methods. The samples found seropositives for the aforementioned TTIs were 756 (2.48%), including 322( 1.06%) blood donors positive for HBsAgs, 392 (1.29%) for HCV antibodies and 49 (0.16%) for HIV Ab/Ag combinations. The remaining 29,714 seronegative donors were then tested by NAT minipool. Nucleic amplification testing on these seronegative samples showed an overall NAT yield of 1:2285. Ten (0.03%) donors tested positive for HBV DNA making HBV NAT yield 1:2971, while only three (0.01%) were positive for HCV DNA making HCV NAT yield 1:9904. No donor was found positive for HIV by NAT testing.

Malaria screening revealed as little as five reactive blood donors (0.02%) while 228 (0.75%) donors were found positive for syphilis.

The frequency of co-infections remained low with only five (0.02%) donors coinfected with HCV and HBV, two (0.01%) with HBV and HIV, two (0.01%) with HBV and syphilis and three (0.01%) with syphilis and HIV.

The prevalence of each TTIs in blood donors is illustrated in Table 1.

**Table 1:** Frequency of Transfusion Transmitted Infections in Blood Donors.

Transfusion Transmitted Infection	Frequency (%)
Hepatitis B	1.09
Hepatitis C	1.30
HIV	0.16
Syphilis	0.75
Malaria	0.02

## Discussion

Window period of an infection is the period between the onset of the infection and the appearance of detectable viral markers in the blood. NAT detects viral ribonucleic acid (RNA) or deoxyribonucleic acid (DNA) in the blood. As it is a highly sensitive technique which involves amplification of targeted parts of the viral genome, the window period of an infection is significantly reduced. The estimated shortening of the window period by NAT is 22 to 11 days for HIV, 59 to 25-30 days for HBV and 70-12 days for HCV. This has resulted in residual risk reduction of transmitting infectious blood components [3]. In nucleic acid testing, the blood samples can be pooled together in a batch of six or eight for testing (mini-pool-NAT/MP-NAT), or tested individually (individual donor-NAT/ID-NAT). MP-NAT is less expensive, but various studies have established the superiority of ID-NAT over MP-NAT in detecting viral markers [4]. As the viral nucleic acid concentration gets diluted in MP-NAT, the sensitivity of NAT is also decreased. Also, if a pool is found reactive, resolution of the entire pool is needed to identify the single reactive sample. This is more time consuming, leading to a delay in the reporting of results. Some scientific models estimate that NAT reduces the infectious window period by 35-91% for HIV one, HCV and HBV with individual donation testing, while only 17-87% with mini-pool (pools of 16) nucleic acid testing [5,6].

NAT yield denotes NAT positive but seronegative blood sample. Most resource-limited countries exhibit a high prevalence rate of TTIs. These countries are likely to yield a significant number of window period donations. Therefore, NAT screening of TTIs in these populations is most cost-effective as compared to the developed world where the TTIs prevalence is low [7]. In a study conducted in North India in 2012 [8] the HBV NAT yield was 1:2972 donations which was much higher than in studies conducted in Western Europe and the USA, where the reported prevalence was around 1:600,000–1:350,000 donations. However, it is much similar to the HBV NAT yield (1:2971) and overall NAT yield (1:2285) of our study. This may be attributed to the high prevalence of infectious diseases in both India and Pakistan as compared to the western world.

A study was conducted from 2015 to 2016 in the blood bank of a tertiary care hospital in southern Pakistan. The results showed serological tests showing 1.71% donors reactive for hepatitis B, 2.12% for hepatitis C and only 0.08% for human immunodeficiency virus. The frequency for syphilis and malaria was 1.69% and 0.01%, respectively. Later, all seronegative samples were subjected to NAT with an overall NAT yield of 1:1143 blood donations. HBV NAT yield was 1:1600 and HCV-NAT yield 1:4000 [9]. As the results of this study showed an overall higher prevalence of TTIs than our study, the NAT yield was also higher.

The NAT yield in our study (1 in 2285) is comparable to a study conducted on 56,772 blood donors at Armed Forces Institute of Transfusion located in Rawalpindi (northern Pakistan) which showed an overall NAT yield of 1 in 2016 donations, HBV NAT yield, 1:2367 and HCV NAT yield, 1:13,6096 [10].

Another study conducted in Children Hospital and Institute of Child Health in Lahore, Pakistan from 2015 to 2016 [11] demonstrated a much higher rate of frequency of TTIs in donors (7.94%) than our study (3.27%). High prevalence of TTI markers (5.46%) in blood donors was also seen in a study conducted in another tertiary care hospital of the same city [12]. However, both aforementioned studies showed HCV infection was the most common amongst blood donors (3.75%) [11] and (2.62%) [12], similar to our study (1.30%). The lower prevalence of HBV infection compared to HCV infection can be explained by the increasing use of hepatitis B immunization in Pakistan, in the recent years.

Results of a study performed at National Institute of Blood Disease and Bone Marrow Transplantation in Karachi from 2013 to 2015 [13] also revealed a much higher rate of donors found reactive for a single TTI (5.8%) and multiple TTIs (0.35%) than our study of 3.27% and 0.04%, respectively. However, the prevalence of malaria (0.07%) in donors was much closer to our figures of 0.02%. This is in contrast to other epidemiological data of the region as Pakistan is a malaria endemic country. A study conducted in 2011 across Pakistan on the prevalence of plasmodium infections revealed a prevalence of an alarming 6.6%, including as high as 13.9% in Zhob, Balochistan. The overall prevalence reported in Islamabad was 4.6% [14].

Syphilis transmission is another major threat to blood recipients due to the emerging annual increase in its incidence. According to WHO, there are 5.6 million new infections of syphilis each year [15]. In our study prevalence of syphilis among blood donors was found to be 0.75% which is much lower than another study conducted in our region which reported 1.6% prevalence in female blood donors and 3.1% prevalence among male blood donors [16]. In Karachi, screening 16,602 blood donors showed syphilis is positive in 2.1% blood donor population [13]. The high prevalence of syphilis in blood donor population is alarming as most of the blood donation centers in Pakistan do not routinely perform syphilis screening and donors are reluctant to declare the disease during pre-donation interview due to the taboo associated with sexually transmitted infections.

Overall, our study has shown a much lower frequency of TTIs in blood donors. This may be attributed to stringent screening of donors by declaring high-risk behaviour in a comprehensive questionnaire before donation. Some limitations of our study included the exclusion of people below 18 years and over 60 years of age, less than 50 kg, and below pre-defined hemoglobin cut off values. As chronic infections including hepatitis and HIV may decrease weight and hemoglobin levels, some infected donors may have been excluded by default. Also, our blood bank does not accept donations from paid donors, who are more likely to be infected than nonremunerated donors because of frequent needle pricks.

Nucleic acid amplification test also detects samples that are false positive by serological methods. In a Malaysian study involving 1388 donor samples, 1.37% samples were reactive on standard serology methods but non-reactive by NAT. On subsequent confirmatory serological tests, these samples were found to be false positive [17]. This is of immense importance as deferral of uninfected donors or blood products can lead to wastage of resources. As our study did not test the seropositive samples, the benefit of NAT testing in excluding false positive results could not be employed.

The NAT yield in our study reiterated the need for NAT and serological testing both for viral TTIs. However, considering Pakistan is a relatively resource-limited country issues including the high cost for NAT equipment, training of staff, reagent procurement and maintenance of machines, need to be weighed against the benefits.

## Conclusions

Donor screening plays a vital role in improving transfusion safety. As a hemovigilance activity, upgraded and standardized screening practices must be implemented to prevent the spread of infectious diseases. Due to the high incidence of TTIs in Pakistan, NAT may be effective in identifying window period infections. However, due to less centres offering NAT screening in Pakistan, there is not enough data to compare the advantages with the cost and infrastructure required. Meanwhile, emphasis needs to be placed on the education of masses to prevent the acquisition of these infections by avoiding high-risk behaviour.
